# Prevalence and Associated Factors of Cognitive Dysfunction Among Middle-Aged Type 2 Diabetes Mellitus Patients in South India: A Cross-Sectional Study

**DOI:** 10.7759/cureus.88747

**Published:** 2025-07-25

**Authors:** Dhrubajyoti J Debnath, Siva Santosh Kumar Pentapati, Hitesh Babu Sarihaddu

**Affiliations:** 1 Department of Community and Family Medicine, All India Institute of Medical Sciences, Mangalagiri, Mangalagiri, IND; 2 Department of Preventive Oncology, Mahamana Pandit Madan Mohan Malaviya Cancer Centre and Homi Bhabha Cancer Hospital, Tata Memorial Centre, Varanasi, IND; 3 Department of Preventive Oncology, Homi Bhabha National Institute, Mumbai, IND

**Keywords:** cognitive dysfunction, diabetes, middle age, moca, risk factors

## Abstract

Introduction

Cognitive dysfunction significantly influences people's day-to-day functioning by impairing their capacity for thought, memory, and problem-solving. The major risk factor for cognitive dysfunction is diabetes mellitus. The middle-aged population is the main breadwinners of the family, yet the most neglected when it comes to health. With our study, we evaluated the prevalence of cognitive dysfunction among middle-aged patients with diabetes mellitus and identified the risk factors associated with it.

Subjects and methods

We conducted a study involving 329 middle-aged patients (aged 40-69) with type 2 diabetes mellitus attending the outpatient department (OPD) of community and family medicine (CFM), general medicine, and endocrinology at a medical college located in the southern region of India. We assessed cognitive dysfunction in these patients using a standardized cognitive screening tool. Then, an interview schedule was taken, which contained information on sociodemographic variables and anthropometry. Recent reports of investigations, viz., fasting blood glucose, glycated hemoglobin (HbA1C) values, and body mass index (BMI) were also recorded. Institutional ethics committee approval was obtained prior to the start of the study. Written informed consent was obtained from all the participants.

Results

The mean (standard deviation (SD)) age of the participants was found to be 51 ± 5.6 years. Among the 329 middle-aged participants with type 2 diabetes mellitus, there were 162 male participants (49.24%) and 167 female participants (50.76%). The prevalence of cognitive dysfunction among middle-aged patients with type 2 diabetes mellitus in the current study was 48.6%, with a 95% confidence interval (CI) of 43.2%-54%. In contrast, higher years of completed education, better socioeconomic status class, and receiving two or more doses of COVID-19 vaccines were associated with lower odds of cognitive dysfunction, indicating a protective effect.

Conclusions

It is crucial for India to prioritize cognitive health, invest in research and education, and develop comprehensive strategies to address this growing issue among the middle-aged diabetic population. Focused efforts of prevention, screening, and managing cognitive impairment should be started.

## Introduction

Cognitive dysfunction refers to deficits in attention, short-term memory and working memory, verbal and nonverbal learning, problem-solving, visual and auditory processing, processing speed, and motor functioning [1]. Cognitive dysfunction and cognitive impairment are commonly used interchangeably. A systematic review on adults aged 50 years and above suggested that the burden of cognitive impairment ranged between 5.1% and 41%, with a median of 19% (interquartile range (IQR): 12%-24.9%) [2]. In India, with a population of over 1.4 billion people, the impact of cognitive dysfunction could be widespread. The difficulty of cognitive dysfunction is not only felt by individuals and their families but also extends to the healthcare system and society as a whole. Additionally, the lack of awareness and resources for diagnosis, treatment, and support further compounds the burden.

Globally, 529 million people (95% uncertainty interval (UI): 500-564), yielding a global age-standardized prevalence of 6.1%, were living with diabetes mellitus [3]. Cognitive dysfunction is widely studied among diabetes mellitus patients belonging to elderly age groups around the world and India. Hypoglycemia and hyperglycemia are known to cause cognitive dysfunction; however, the underlying pathophysiology is not well understood [4].

The Montreal Cognitive Assessment (MoCA) test is an instrument useful for the domain of global cognitive delay, assessing different cognitive domains such as attention and concentration, executive functions, memory, language, visuoconstructional skills, conceptual thinking, calculations, and orientation. It is a straightforward, independent cognitive screening instrument with high sensitivity. It has outstanding test-retest reliability, covers significant cognitive domains, and has both positive and negative predictive values for mild cognitive impairment (MCI) [5].

Literature suggests that the relationship between type 2 diabetes mellitus and cognitive dysfunction among the elderly group is well established, but scarcity exists for the middle-aged population.

This knowledge gap prompted the present study to evaluate the prevalence of cognitive dysfunction among middle‑aged patients with type 2 diabetes mellitus and to identify its associated risk factors.

The specific objectives were as follows: to determine the prevalence of cognitive dysfunction in middle-aged patients with type 2 diabetes mellitus, to determine the association between sociodemographic factors associated with cognitive dysfunction using multivariable logistic regression, and to evaluate the effect of COVID-19 vaccination (≥2 doses) on the odds of cognitive dysfunction.

## Materials and methods

Study population

We conducted a cross-sectional study among middle-aged patients aged between 40 and 69 years with type 2 diabetes mellitus attending the outpatient department (OPD) of community and family medicine (CFM), general medicine, and endocrinology at a medical college located in the southern region of India, Andhra Pradesh. Sample size was calculated using Cochran's sample size formula (Z2pq/d2), where Z was a standard normal deviate that corresponds to 95% confidence level (1.96); p was the expected prevalence of cognitive dysfunction among middle-aged type 2 diabetes mellitus patients, taken as 69% from a study done in Punjab, India [6]; q is (100 - p); and d was absolute precision of 5%. The required sample size for the study was 329. Cognitive dysfunction in these patients was assessed using the Montreal Cognitive Assessment (MoCA) score [5]. The MoCA questionnaire can be used without copyright issue [7]. The MoCA test can be used for educational purposes without permission, but written permission and a licensing agreement are required if the research is funded by a commercial entity or pharmaceutical company [8]. The present study was not funded by a commercial entity or a pharmaceutical company. This study was conducted by the principal investigator in the Bachelor of Medicine and Bachelor of Surgery (MBBS) course to promote research aptitude in the education curriculum. This study was carried out as a part of undergraduate medical education.

The data collection period spanned from December 5, 2023, to February 5, 2024. Patients who denied consent and patients with type 1 diabetes mellitus, gestational diabetes mellitus, and seriously ill cases of type 2 diabetes mellitus were excluded from the study. Patients with evidence of head injury, infections, known history of neurological disease, chronic kidney disease stage 5, hepatocellular carcinoma, and significant hearing or visual impairment that could interfere with cognitive testing were also excluded from the study.

Study procedure

Institutional ethics committee approval was obtained prior to the start of the study. After obtaining written informed consent from eligible participants, their global cognitive function was assessed using the Montreal Cognitive Assessment (MoCA) test. The entire cognitive assessment took approximately 30-40 minutes to complete. A MoCA test score of 25 and below was considered indicative of cognitive dysfunction. Subsequently, an interview schedule was taken, which contained information on sociodemographic variables, COVID-19 history, COVID-19 vaccination (self-reported), related information, and anthropometry. Recent reports of investigations, viz., fasting blood glucose and glycated hemoglobin (HbA1C) values, were also recorded.

Data analysis

Survey data was entered into Microsoft Excel 2010 (Microsoft Corp., Redmond, WA) and then imported into Stata/MP-Parallel Edition 17.0 (StataCorp LLC, College Station, TX) for statistical analysis. Continuous variables were expressed as mean ± standard deviation (SD) for data with normal distribution or median with interquartile range (IQR) for data with skewed distribution. Nominal data were expressed as numbers and percentages. Correlation between the scores of the tests used for cognitive assessment was measured using paired-wise correlation, and scattergrams were plotted. A MoCA score of 25 was taken as a cutoff, and a new outcome variable (cognitive dysfunction) was created with binary response (yes or no). This variable was taken as outcome variable for the regression analysis. A bivariate logistic regression analysis was carried out first between the outcome variable and most of the variables in the data. Then, a multivariate logistic regression analysis was done to estimate the association between the outcome variable and exposure variables; variables with a p-value of 0.2 or less in the bivariate logistic regression analysis were included in the multivariate logistic regression analysis. For all analyses, a p-value of less than 0.05 was considered statistically significant.

## Results

As seen in Table 1, 77 male participants and 83 female participants, i.e., 160 (48.6%) out of 329 middle-aged type 2 diabetes mellitus patients, were found to have cognitive dysfunction (MoCA score of 25 and below). Thus, 48.6% of the participants in the present study had cognitive dysfunction.

**Table 1:** Distribution of cognitive dysfunction by MoCA score (N = 329) MoCA: Montreal Cognitive Assessment

MoCA score	Cognitive dysfunction	Male (number (%))	Female (number (%))	Total (number (%))
25 and below	Yes	77 (47.53)	83 (49.7)	160 (100)
26 and above	No	85 (52.47)	84 (50.3)	169 (100)

A detailed distribution of the sociodemographic and important study variables among the participants is presented in Table 2. The mean (SD) age of the study participants was found to be 51 ± 5.6 years. Among the 329 participants, there were 162 male participants (49.24%) and 167 female participants (50.76%). The median (IQR) of completed years of education of the participants was 10 (5-15). The median (IQR) monthly family income of the participants was INR 30,000 (INR 20,000-40,000). According to the modified BG Prasad scale, the majority of the study participants (169 (51.36%)) belonged to the upper middle class, and 131 (39.82%) participants belonged to the upper class [9]. The mean (SD) body mass index (BMI) of the participants was 27.6 (3.66) kg/m^2^. As per the consensus statement for the diagnosis of obesity, abdominal obesity, and metabolic syndrome for Asian Indians [10], only 37 (11.25%) participants had a normal BMI (18-22.9 kg/m^2^). The mean (SD) fasting blood glucose level of the participants was 166.9 ± 29.1 g/dL. The mean (SD) HbA1C value of the participants was 8.4 ± 1.52. Of the participants, 205 (62.3%), 53 (16.1%), 80 (24.3%), and 42 (12.8%) were suffering from hypertension, had a history of tobacco exposure, had a history of alcohol intake, and had COVID-19 infection in the past, respectively. Raised blood pressure and increased age were associated with higher odds of cognitive dysfunction. Higher years of education, better socioeconomic status class, and receiving two or more doses of COVID-19 vaccines were associated with lower odds of cognitive dysfunction, i.e., these variables had a protective effect against cognitive dysfunction.

**Table 2:** Distribution of sociodemographic and important study variables among the participants (N = 329) SD: standard deviation, IQR: interquartile range, INR: Indian rupee, BMI: body mass index, HbA1C: glycated hemoglobin, COVID-19: coronavirus disease 2019

Variable	Distribution
Age (mean ± SD) (years)	51 ± 5.6
Sex (number (%))	
Male	162 (49.24)
Female	167 (50.76)
Completed years of education (median (IQR))	10 (5-15)
Monthly family income (median (IQR)) (INR)	30,000 (20,000-40,000)
Socioeconomic status (number (%))	
Lower middle	5 (1.52)
Middle	24 (7.29)
Upper middle	169 (51.37)
Upper	131 (39.82)
Height (mean ± SD) (cm)	162.7 ± 11.67
Weight (mean ± SD) (kg)	73.7 ± 11.38
BMI (mean ± SD) (kg/m^2^)	27.6 ± 3.66
BMI categories	
Underweight (BMI < 17.9 kg/m^2^)	1 (0.3)
Normal (BMI = 18-22.9 kg/m^2^)	37 (11.25)
Overweight (BMI = 23-24.9 kg/m^2^)	29 (8.81)
Obese (BMI ≥ 25 kg/m^2^)	262 (79.64)
Systolic blood pressure (mean ± SD) (mmHg)	135.3 ± 12.72
Diastolic blood pressure (mean ± SD) (mmHg)	82.9 ± 7.14
Fasting blood glucose (mean ± SD) (g/dL)	166.9 ± 29.1
HbA1C (mean ± SD)	8.4 ± 1.52
Hypertension (number (%))	
Yes	205 (62.3)
No	124 (37.7)
Tobacco exposure (number (%))	
Yes	53 (16.1)
No	276 (83.9)
Alcohol intake (number (%))	
Yes	80 (24.3)
No	249 (75.7)
COVID-19 infection in the past (number (%))	
Yes	42 (12.7)
No	287 (87.3)
COVID-19 vaccination history (number (%))	
Less than 2 doses	33 (10)
2 doses	244 (74.2)
2 doses + booster dose	52 (15.8)

Table 3 presents the factors associated with cognitive dysfunction among the study participants.

**Table 3:** Factors associated with cognitive dysfunction (N = 329) COR: crude odds ratio, CI: confidence interval, AOR: adjusted odds ratio, SBP: systolic blood pressure, DBP: diastolic blood pressure, BMI: body mass index, COVID-19: coronavirus disease 2019, HbA1C: glycated hemoglobin

Variable	Number (%)	COR	95% CI	p-value	AOR	95% CI	p-value
Age categories							
45 years or less	63 (19.15)	Reference	-	-	-	-	-
46-50 years	96 (29.18)	2.29	1.03-5.13	0.04	2.2	0.79-6.16	0.13
51-55 years	96 (29.18)	10.12	4.56-22.43	<0.01	7.17	2.50-20.55	<0.01
56 years or more	74 (22.49)	19.21	8.02-46.02	<0.01	10.31	3.09-34.36	<0.01
Sex							
Male	162 (49.24)	Reference	-	-	-	-	-
Female	167 (50.76)	0.92	0.59-1.41	0.69	-	-	-
Socioeconomic status							
Social class III (2544-4239)	48 (14.59)	Reference	-	-	-	-	-
Social class II (4240-8479)	183 (55.62)	0.45	0.23-0.9	0.02	0.25	0.10-0.64	<0.01
Social class I (≥8480)	98 (29.79)	0.18	0.09-0.39	<0.01	0.23	0.08-0.65	<0.01
Education years categories							
5 years or less	104 (31.61)	Reference	-	-	-	-	-
6-10 years	81 (24.62)	0.23	0.12 -0.44	<0.01	0.45	0.20-1.03	0.06
11 years or more	144 (43.77)	0.06	0.03-0.11	<0.01	0.16	0.07-0.35	<0.01
Raised blood pressure (SBP > 140 mmHg or DBP > 90 mmHg or both)							
No	214 (65.05)	Reference	-	-	-	-	-
Yes	115 (34.95)	4.92	2.99-8.1	<0.01	2.72	1.39-5.30	<0.01
BMI categories							
Normal and underweight	38 (11.55)	Reference	-	-	-	-	-
Overweight	29 (8.81)	1.23	0.47-3.25	0.68	-	-	-
Obese	262 (79.64)	0.91	0.46-1.80	0.79	-	-	-
History of tobacco usage							
Yes	53 (16.1)	1.76	0.97-3.21	0.06	1.07	0.37-3.07	0.90
No	276 (83.9)	Reference	-	-	-	-	-
History of alcohol intake							
Yes	80 (24.3)	1.40	0.84-2.32	0.19	0.87	0.34-2.21	0.76
No	249 (75.7)	Reference	-	-	-	-	-
History of hypertension comorbidity							
Yes	205 (62.31)	2.65	1.67-4.21	<0.01	0.98	0.47-2.02	0.95
No	124 (37.69)	Reference	-	-	-	-	-
Years of suffering from diabetes							
5 years or less	146 (44.38)	Reference	-	-	-	-	-
6-10 years	122 (37.08)	3.57	2.14-5.93	<0.01	1.54	0.75-3.19	0.24
More than 10 years	61 (18.54)	7.59	3.83-15.05	<0.01	1.72	0.65-4.56	0.28
History of COVID-19 infection in the past							
Yes	42 (12.7)	Reference	-	-	-	-	-
No	287 (87.3)	1.19	0.62-2.27	0.60	-	-	-
COVID-19 vaccine history							
Less than 2 doses	33 (10)	Reference	-	-	-	-	-
2 doses	244 (74.2)	0.12	0.04-0.34	<0.01	0.22	0.06-0.8	0.02
2 doses + booster dose	52 (15.8)	0.08	0.02-0.26	<0.01	0.19	0.04-0.83	0.03
Glycemic control achieved (HbA1C < 7)							
Yes	40 (12.16)	Reference	-	-	-	-	-
No	289 (87.84)	3.24	1.53-6.87	0.02	1.48	0.55-4.03	0.44

## Discussion

In the current study, the prevalence of cognitive dysfunction among middle-aged type 2 diabetes mellitus patients was 48.6%, with a 95% confidence interval (CI) of 43.2%-54%. Various studies across the country assessing the prevalence of cognitive dysfunction in middle-aged type 2 diabetes mellitus patients using the MoCA test for cognitive assessment reported a prevalence ranging from 23.9% to 92% [6,11-14]. Studies conducted outside India also reported a prevalence similar to studies done in India [15,16].

This study did not find any association between COVID-19 infection in the past and the participants' current status of cognitive function. This study found that participants who had received two doses or two doses and a booster dose of COVID-19 vaccine had lower odds of cognitive dysfunction compared to participants who had received one dose or did not receive a COVID-19 vaccine. Studies conducted in Wuhan, China, reported that COVID-19 patients had lower Telephone Interview of Cognitive Status-40 (TICS-40) (Chinese version) scores and higher Informant Questionnaire on Cognitive Decline in the Elderly (IQCODE) scores [17]. Another study conducted in Hangzhou, China, reported that COVID-19 patients had a lower correct number on Continuous Performance Test 2 and Continuous Performance Test 3 compared with controls (9.83 ± 1.93 versus 8.21 ± 1.90, P = 0.002) [18]. Study done in Madrid, Spain, reported that compared to healthy controls, patients with COVID-19 showed worst scores in the recall and recognition trials of the Figural Memory Test (FGT), Inhibition Test, N-Back Verbal (NBV), and Trail Making Test (TMT-A and TMT-B), and several visual tasks of the WAF Battery [19]. The difference in the results of the present study compared to other studies might be because of the different methodological aspects such as study settings, study population, and COVID-19 vaccine policy.

The present study found that higher years of completed education and better socioeconomic status class were associated with lower odds of cognitive dysfunction, i.e., they had a protective effect. This is similar to the findings in other countries. Clouston et al. reported that healthy life expectancy without cognitive impairment increased with education [20]. Alley et al. found that higher education levels were consistently associated with higher cognitive function and lower risk of dementia [21]. In their study, Zhang et al. discovered that those with low economic status had a higher prevalence of cognitive impairment in the elderly age group [22].

Our present study also found that receiving two or more doses of COVID-19 vaccines had a protective effect. In their investigation, Roh et al. discovered a strong correlation between COVID-19 vaccination, specifically mRNA vaccines, and higher rates of moderate cognitive impairment (MCI) and Alzheimer's disease (AD) [23]. The reason may be attributed to different types of COVID-19 vaccines used worldwide.

The strengths of our study were as follows: it was conducted on the most neglected but the most important group, i.e., middle-aged population; it calculated sample size prior to the study and achieved the required power; and it used a well-studied and validated scale, i.e., the MoCA scale. The main limitation of the study was that it was a hospital-based study, so the study results may not be generalized to the real-world population.

## Conclusions

Our study found that around half of the middle-aged type 2 diabetes mellitus patients were suffering from cognitive impairment. The present study found that hypertension and advancing age were risk factors, whereas higher years of completed education, better socioeconomic status class, and receiving two or more doses of COVID-19 vaccines had a protective effect against developing cognitive dysfunction. The study throws significant light on cognitive impairment and the factors associated with it in the middle-aged type 2 diabetes mellitus population in the southern region of India, especially after the COVID-19 pandemic. It is crucial for India to prioritize cognitive health, invest in research and education, and develop comprehensive strategies to address this growing issue among the middle-aged diabetic population. Focused efforts of prevention, screening, and managing cognitive impairment should be started.
